# Understanding Preventive Health Behavior: A Mixed-Methods Study to Explore Factors Influencing the Practice of Breast Self-Examination Among Indian Medical Students

**DOI:** 10.7759/cureus.62151

**Published:** 2024-06-11

**Authors:** Himabindu Reddy, Abhishek Joshi, Shiv H Joshi, Vatsala Gupta, Ashok M Mehendale

**Affiliations:** 1 Department of Community Medicine, Jawaharlal Nehru Medical College, Datta Meghe Institute of Medical Sciences, Wardha, IND; 2 Department of Epidemiology and Public Health, World Health Organization, National Tuberculosis Elimination Programme Technical Support Network, Agra, IND

**Keywords:** knowledge-behavior gap, early detection of cancer, breast cancer screening barriers, enablers and barriers, mixed methods research, low income countries, awareness of breast cancer, screening of breast cancer, breast self-examination (bse)

## Abstract

Introduction: Breast cancer (BC) is among the most prevalent oncological cases in the world, and the global burden of the disease is expected to rise further in the coming years. Strategies aiming at early diagnosis, backed by research and a well-trained healthcare cadre, can aid low- and middle-income countries (LMIC) in tackling the possible cancer-caused strain on healthcare systems. Our study aimed to evaluate the level of knowledge of medical students concerning BC and explore barriers and facilitators of breast self-examination (BSE).

Methods: A sequential explanatory mixed-methods study approach to better understand factors and beliefs influencing preventive health practice in BSE was conducted among students at a medical college in rural Maharashtra, India. One hundred and two female medical students completed the quantitative phase, and 15 of them gave in-depth interviews (IDIs) for the qualitative aspect.

Results: Among the participants, 67.6% had good knowledge of risk factors, but only 10% knew the recommendations for BSE, clinical breast examination (CBE), and mammography. We found that being taught BSE by a trusted source and knowing a BC patient were significant facilitators. In contrast, lack of self-efficacy and two fear factors were found to be acting as barriers for BSE, one being the absence of fear of ever getting BC and the other fear of detecting a lump.

Conclusion: This study reveals a gap between knowledge of risk factors and their translation to disease prevention practice. The barriers elicited are modifiable by planning and implementing an appropriate training program covering risk factors and recommending all available screening and preventative modalities. A well-trained medical staff will be instrumental in improving the health status of our community and country.

## Introduction

Cancer is the second-leading cause of death worldwide. A systematic analysis of the global burden of disease from 1990-2015 concluded that the incidence of malignancies will rise in the coming years. If control and prevention strategies are poorly planned and executed, this rise in cases will cause strain on our health systems [1].

Since 2020, breast cancer (BC) has been the most prevalent cancer in the world [2]. While increasing advancements in medical sciences have added to the screening, diagnosing, and treatment options available for BC, the disease is still the leading cause of cancer deaths in females, both in developing and developed nations alike. While industrialized countries have a higher incidence of BC, less developed countries have a higher mortality rate [3]. The International Agency for Research on Cancer estimated 1.67 million new BC cases globally in 2012 and upwards of 0.5 million deaths, of which around 0.1 million deaths occurred in India. The American Cancer Society's (ACS) estimates report that five-year survival rates are 99% for localized BC (Stage 1) and only 6%-22% for regional or metastasized BC (Stages 3-4) [2]. In India, approximately 8% of cases are detected in Stages 1 and 23%-58% in Stages 2 and 3 [4]. These delayed diagnoses can be attributed to a lack of awareness about risk factors, symptoms, and suboptimal BC screenings. The scope of cancer prevention by controlling risk factor exposure has been demonstrated by a fall in cancer incidences with the help of vaccines, tobacco control, and other modifiable risk factors. Breast cancer rates have also shown improvement in nations where screening programs have improved access to diagnosis and treatment [5].

The Global Breast Cancer Initiative (GBCI) put forth three pillars for cancer control operations: health promotion for early detection, timely diagnosis, and comprehensive BC management, with the aim of reducing global BC mortality by 40% by 2040 [6]. The World Health Organization also stresses that screening an asymptomatic target group of seemingly healthy individuals is essential for the early diagnosis of cancer [7]. Breast self-examination (BSE), clinical breast examination (CBE), mammography, ultrasonography, and histopathology are the preventive screening and confirmatory modalities for breast lumps. Breast self-examination is the practice of regularly inspecting and palpating one's breast tissue to familiarize oneself with its normal look and feel so that any rigidity, lump, or swelling can be identified and examined clinically at the earliest. While the American Cancer Society (ACS) guidelines for early detection of BC do not offer guidelines for CBE and BSE, it does recommend women starting in their twenties be familiar with how their breasts look and feel so that they can report and investigate the occurrence of any abnormality at the earliest [8]. While developed nations with widespread mammography programs may debate the harm-benefit of BSE, in low- and middle-income countries (LMIC) with limited health centers and screening facilities, a free-of-cost and easy-to-perform technique like BSE is undoubtedly a valuable tool in the fight against BC.

Aim

Medical students and interns of today are the task force of tomorrow and, hence, a valuable group to assess for factors influencing preventive behavior in BC. It is expected that medical students will, through their coursework, gain knowledge about BC and the practice of BSE and have a positive attitude towards it. Our study aimed to evaluate their level of knowledge concerning BC and BSE practice and explore barriers and facilitators of BSE.

## Materials and methods

We adopted a sequential explanatory mixed-methods study approach with both quantitative and qualitative aspects to better understand the preventive health behavior patterns of educated youth: interns and final-year students at a medical college in Maharashtra, India. The study was approved by the institutional ethics committee, Datta Meghe Institute of Medical Sciences, Wardha, India (approval number: 9085).

One hundred and forty-four participants fit our inclusion criteria of being female, above 18 years old, and medical students 102 completed the quantitative phase (September 2022-December 2022). A structured questionnaire was developed after literature research and refined with input from subject experts in surgery, preventive medicine, and public health. The questionnaire was then piloted with 10 individuals and revised accordingly. The questionnaire had three sections covering personal details, risk factor knowledge about risk factors and screening practices, and the frequency and consistency of BSE. We did not include many questions on attitudes, perceptions, or barriers to BSE, which were explored in the qualitative phase.

**Figure 1:**
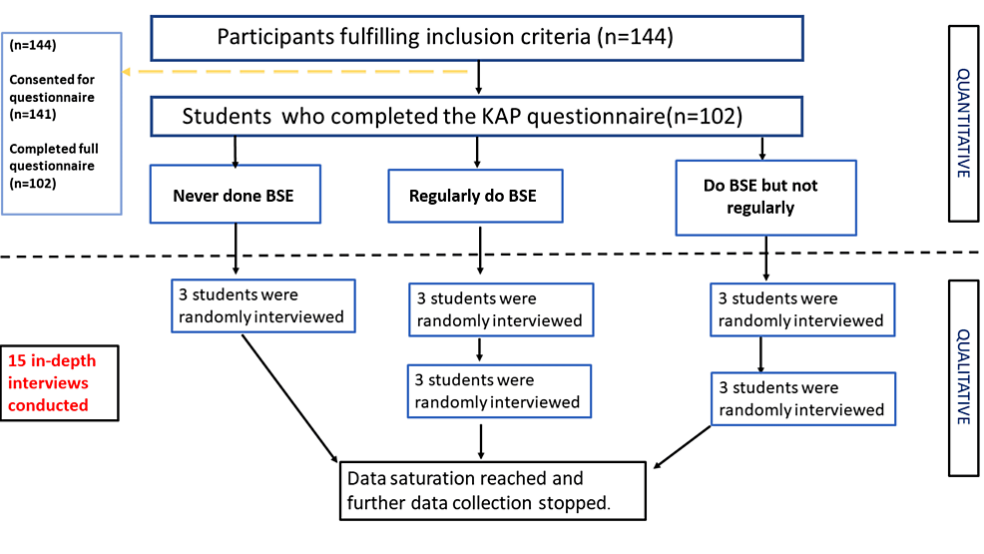
The study methodology flow chart KAP: knowledge, attitudes, and practices; BSE: breast self-examination

The list of all the 102 study participants was used to generate three sub-lists according to their BSE practices. Three individuals from each of these sub-lists were interviewed each round until the point of data saturation. A total of 15 in-depth interviews (IDI) were conducted. Figure 1 depicts the flow of methodology followed in brief.

All the interviews were conducted in person, at a time and place, as per the feasibility of individual participants. An interview guide with scope to ask any relevant question that was not in the guide as the interview evolved to ensure not to miss out on any perspectives of the study participants. We aimed to understand why the participants who regularly performed BSE were doing so, and why others had never performed BSE. All the interviews were recorded as voice notes on the mobile device of the principal investigator. These audio files were transcribed verbatim for analysis.

Data analysis

The collected responses were entered in a Microsoft Excel file (Microsoft Corp., Redmond, WA) imported into RStudio, running on R version 4.1.2 (Posit PBC, Boston, MA), for data cleaning and analysis. The data were explored for duplicates or missing data. The data analysis plan pre-defined that if we have >10% of the data points missing for any of the participants, we will exclude that participant's records from the analysis; for the variables that were categorical or discrete, counts and percentages were reported, whereas for the continuous variables, the mean with standard deviation was reported. The descriptive statistics were reported for the respective sections of the study questionnaire. The association of BSE with sociodemographic variables was explored by performing chi-square tests. A similar analysis was conducted to understand the association between BSE and knowledge of BC risk factors, available screening modalities, and participants' perceptions of BSE.

Thematic analysis was done with transcripts of the in-depth interviews. All four researchers individually read all the transcripts multiple times until they felt they understood everything that the participants wanted to convey. They then conferred and familiarized themselves further with all the viewpoints, and thematic analysis was done to understand factors promoting and inhibiting the practice of BSE among study participants.

## Results

Sociodemographic characteristics

The sociodemographic characteristics are summarized in Table 1. The mean age of our study participants was 23 years (±1.24). While >90% of our participants were Hindu by religion, our study included participants of Muslim, Christian, and Sindhi faiths. Most (71.3%) of the participants belonged to urban households. We wanted to explore if the type of family influences young adults' habits. Participants belonged to varied nuclear families, while 30% were from joint and 27% from three-generational families. Among the participants, 81% were interns, and only 6% were married at the time of the study.

**Table 1:** Sociodemographic features of the study population

Sociodemographic category	Characteristics	n (%)
Age (years)	Mean (SD)	23.78(1.24)
Religion	Hindu	89(88.27%)
	Christian	5(4.8%)
	Muslim	6(5.8%)
	Sindhi	2(1.9%)
Place of residence	Urban	72(71.3%)
	Semi-urban	21(20.8%)
	Rural	8 (7.9%)
Type of family	Nuclear	44(43.6%)
	Joint	30(29.7%)
	Three-generational	27(26.7%)
Designation	Intern	82(81.2%)
	Student	29(28.8%)
Marital status	Unmarried	95(94.05%)
	Married	6(5.95%)

Knowledge about risk factors

After a thorough literature search, we formulated section B of our questionnaire to test the participants' knowledge of breast cancer risk factors. The 18 questions in this section were in the form of close-ended statements, which the participants had to select from the options yes, no, or don't know. We included the opportunity to limit the false positive knowledge score by participants guessing the correct answers by chance. Each correct answer was given a score of one; otherwise, it was 0. Since our study population consisted of medical students, we expected most of them to have a good awareness of the topic. Hence, we kept a score of 75% as the limit for qualifying as having a good level of knowledge. As depicted in Table 2 below, 33 participants showed good knowledge of BC risk factors in our study. We found good awareness among the students about most of the risk factors. Still, awareness was lacking about some risk factors like having dense breasts (57%), nulliparity (51%), not breastfeeding (43%), late menopause (57%), history of hormone replacement therapy (HRT) (47%), and radiation therapy (50%).

**Table 2:** Participants’ knowledge of breast cancer risk factors

Questions/statements to examine knowledge of breast cancer risk factors	Frequency (%) of correct responses
Breast cancer has surpassed cervical cancer as the most common female cancer in India	80(78.4%)
Breast cancer is a communicable disease	98(96%)
Breast cancer is always hereditary	87(85.3%)
Only females are affected by breast cancer	92(90.2%)
Being female increases the risk of breast cancer	87(85.3%)
Dense breasts increase the risk of breast cancer	58(57%)
Early menarche increases the risk of breast cancer	88(86.3%)
Late menopause increases the risk of breast cancer	58(57%)
Late age at first full-term pregnancy (>30 years) increases the risk of breast cancer.	88(86.3%)
Nulliparity increases the risk of breast cancer	52(51%)
Breastfeeding increases the risk of breast cancer	44(43%)
The use of oral contraceptive pills increases the risk of breast cancer.	85(85.3%)
Hormone replacement therapy for the treatment of menopause increases the risk of breast cancer	48(47%)
History of radiation therapy increases the risk of breast cancer	51(50%)
Tobacco use can cause breast cancer	80(78.4%)
Alcohol abuse increases the risk of breast cancer	84(82.4%)
Obesity increases the risk of breast cancer	92(90.2%)
A stressful lifestyle can increase the risk of breast cancer	80(78.4%)
Poor level of knowledge (score <75%)	33(32.4%)
Good level of knowledge (score >75%)	69(67.6%)

Knowledge of breast cancer screening

Section C of our questionnaire dealt with the knowledge of participants about various modalities of BC screening and recommendations about when to start and how frequently to get each screening. Table 3 below shows that participants had poor knowledge about screening modalities and recommendations. Only a fraction of the participants knew the right age to start regular BSE (23.5%) and mammography (26.5%). The recommended frequency to perform BSE, CBE, and mammography was correctly known by 61%, 13.7%, and 10%, respectively.

**Table 3:** Knowledge of screening practices for breast cancer BSE: breast self-examination; CBE: clinical breast examination

Questions/statements to examine knowledge of screening modalities and recommendations	Frequency(%)of correct responses
At what age should BSE be started?	24(23.5%)
How often should BSE be done?	62(61%)
Can CBE replace BSE examination?	38(37%)
How often should you ideally get CBE?	14(13.7%)
At what age should screening mammograms be started?	27(26.5%)
How often do you need a mammogram?	10(9.8%)
Can a chest X-ray be done instead of a mammogram?	38(37%)

Correlates of practicing BSE at least once and practicing BSE regularly

As shown below in Table 4, factors significantly associated with regular practice of BSE included type of family (0.03), knowledge of risk factors (0.01), and being taught the technique of BSE (0.01-0.03). Moreover, we found that participants who were introduced to the concept of BSE by healthcare staff (p = 0.003) were significantly more likely to do BSE at least once than those who learned about BSE from other means.

**Table 4:** Factors associated with the practice of breast self-examination (BSE)

Factor	Categories	Ever done BSE: Yes(83); No(19)		Chi-square & p-value	Regularly do BSE: Yes(28); No(74)		Chi-square & p-value
Religion	Hindu	74	15	5.75 & p=0.12	26	64	0.96 & p=0.8
	Christian	3	2		1	4	
	Muslim	3	3		1	5	
	Sindhi	2	0		0	1	
Area of residence	Urban	60	13	2.19 & p=0.13	24	49	3.7 & p=0.05
	Rural	23	6		4	25	
Type of family	Nuclear	36	9	0.84 & p=0.65	18	27	6.94 & p=0.03
	Joint	26	4		5	25	
	Three-generational	21	6		5	22	
Family history of breast cancer	Yes	12	2	0.006 p=1.0	4	10	0.0 & p=1.0
	No	71	17		24	64	
Knowledge of breast cancer risk factors	Yes	29	4	0.8 & p=0.2	24	45	4.6 & p=0.01
	No	54	15		4	29	
Awareness about BSE	Yes	83	16	-	28	71	-
	No	0	3		0	3	
Introduced to BSE by friends & family	Yes	58	11	0.54 & p=0.41	16	53	1.34 & p=0.23
	No	25	8		12	21	
Introduced to BSE by healthcare staff	Yes	79	13	9.6 & p=0.003	27	65	0.86 & p=0.27
	No	4	6		1	9	
Introduced to BSE by multimedia	Yes	58	10	1.36 & p=0.18	15	53	2.22 & p=0.1
	No	25	9		13	21	
Were you taught the BSE technique?	Yes	81	15	6.6 & p=0.01	28	68	-
	No	2	4		0	6	
BSE technique taught by friends & family	Yes	45	4	4.2 & p=0.03	7	37	6.3 & p=0.01
	No	36	11		21	31	
BSE technique taught by healthcare staff	Yes	59	5	7.2 & p=0.006	23	41	4.2 & p=0.03
	No	22	10		5	27	
BSE technique taught by a faculty/teacher	Yes	17	8	5.29 & p=0.021	16	55	5.8 & p=0.01
	No	64	7		12	13	

Thematic analysis

The interviews lasted between 15- 25 minutes with a mean duration of 18 minutes. Themes were extracted around two pre-determined areas which were barriers and facilitators. Some representative quotes from participants have been recounted in Table 5.

**Table 5:** Themes and representative quotes isolated from analysis of participant in-depth interviews BSE: breast self-examination; PG: paying guest

Pre-determined Themes	Themes extracted by the analysis of IDIs	Representative quotes
Barriers: factors inhibiting BSE	Fear of breast cancer	“I had a false scare once. I had just started BSE monthly and had done it for a few months when I thought I felt a lump. I was scared to even go show it to the doctor. I started checking almost every two days to see if it was still there or if it was gone. Finally, when I went to the doctor, she checked and said there was no lump, and then after that, I somehow became hesitant about BSE. I still do it occasionally, once every six months or so, but not regularly. I know I should do it monthly; I'll try starting it this month."
		“There is a fear in my mind that, “Oh my god, what if I do it and I find a lump... or if I find anything abnormal?"
		“I have no family history, so I believed that I didn’t stand any chance for that. But now that I am saying it out loud, I am realizing that I have learned that it can occur even without history; the lifestyle risk factors also cause it, I know, but I never thought seriously about it until now. I will learn and start it this month."
	Discomfort or lack of ease about one's own body	“Recently, one month ago, I was feeling a little bulkier on one side, and I wondered if it was a lump or just a subjective feeling, so I asked my friend to examine me, but my friend was not comfortable. She told me that this is not the age where anything like this can happen, and you are too young for it, and I should stop thinking about it. ”
		"Even though we belong to the medical profession, personally, I am very shy about doing such a thing. And no person in my circle really stressed that this was important or helpful. I mean, I read it in books, but that didn't become a push to try it."
		“To be honest, ma'am, I have never had this discussion before; I never thought about why I am not practicing BSE. I did learn about it in class, but it never registered that I have also turned 20, and I myself also need to start this screening practice."
		“Even after being on the medical side, I think not many know how to exactly do it correctly, and maybe the gravity of the situation that this can happen to anyone hasn’t set in."
		“I have not talked about this so freely. I think I had some thoughts of doing it after reading about it, but I was not understanding how to begin the examination or what is the right feel. Then I didn't know whom to approach, so I just suppressed the thought and let it go."
		“I think it would have helped if she had demonstrated. The session did make us aware, but it didn’t do anything to make us comfortable with our bodies. The instructor just showed some pictures on slides and quickly moved on. I remember having some doubts about the technique at the time, but I hesitated to ask, and then slowly the thought of asking my doubt and starting the practice regularly also dropped from my mind.”
Facilitators: factors enabling BSE	Proper instruction in the technique of BSE	“In our college, there were some meetings conducted for the girls, especially about menstrual hygiene practices, breast cancer, cervical cancer, and the safety of STDs. They used to teach that. We were just introduced to the thought of doing it, which was important.
		“In one of our clinical postings, a PG resident had explained to us about the procedure, and she also demonstrated the procedure on a patient; in fact, she also told us to try it on ourselves and also urged us to try it out on our friends as well. She saw us looking hesitant, and I don’t remember exactly what she said, but the gist was that there is nothing shameful or need for secrecy about BSE, and we should be comfortable with our body parts and do what is recommended to stay safe. She told us we are lucky that we are in a field where we can openly communicate about any body part, and so we should always have some open peer discussions and support each other in cultivating healthy practices. I remember going back to the hostel and giving this advice to my circle who were not posted with us."
	Support from other women in their circle	“Basically, it comes from my family. My mother is of the thought that what can be prevented should be prevented." While I was in college, she took me and my elder sister to get our HPV vaccinations. She also got vaccinated with us. So, she always stresses that prevention is better. She was the one who taught both of us about BSE and stressed the importance of the importance of making it a regular practice. So I learned at home from my mother and my sister."
		“I have seen one of my mom’s friends die of breast cancer, diagnosed so late that it was incurable. She died in two months. My friend and I have always been careful after that. We keep track from time to time that we are screening ourselves regularly."
		“I learned first from my mother and then from the hospital, both times by actual demonstration. So I am quite comfortable doing and teaching others also.”
		“It's already a habit for me; I guess I should be thankful that I get periods regularly every month, so they are like my prompts. On the last day of the cycle, I do BSE to check for changes and lumps. This was the way my sister had asked me to do it when she first taught me. She used to check on me initially, asking if I had done it or not, and in a few months, it became a natural monthly routine.”

Barriers

Among all the participants who were either not at all practicing BSE or participants who were irregular in their practice of BSE, we identified some common factors, which can be grouped under the two themes given below.

Fear of breast cancer: Among our 15 total interviewees, six mentioned fear as one of the factors influencing their behavior. While two of them expressed a fear of receiving a diagnosis, four others cited a lack of fear of BC as a barrier to making BSE a regular practice. The participants acknowledged that they understood most of the lumps may be benign and that finding a lump at the earliest is better for treatment. They confessed that somewhere the fear of actually discovering a lump and getting diagnosed with a tumor was keeping them from practicing BSE regularly, and for some, it was because they believed there was no chance of them ever getting BC. A few representative quotes are listed in Table 5.

Discomfort and lack of ease about one's own body: A common recurring reason cited by all three who never did BSE was a sense of discomfort about the practice. They had awareness about the practice, but they didn't practice either because they didn't receive the required support and encouragement from their peers and instructors about the importance of being aware of one's own breast's normal appearance and feel or because they were unsure about what they were looking for while palpating. They lacked a sense of normalcy and confidence that they could perform the technique easily, and so the theory they knew didn't translate to practice because of that doubt.

Facilitators

Proper instruction in the technique of BSE: The participants who regularly practice BSE said that a major reason why they formed the habit of doing BSE monthly was that they had received a good initiation to the practice, which gave them confidence about examining themselves smoothly and naturally without hesitancy.

Support and encouragement from other women in the family or friend circle: Having female influences like mothers, elder sisters, or friends who are conscious of their health and comfortable discussing these topics with each other proved to have a positive impact on many of the participants and helped them form the habit of doing BSE monthly.

## Discussion

For BC, while BSE alone might have a limited role in reducing mortality, studies done among LMICs have shown it to improve health outcomes. A community-based trial done in Iran analyzed the five-year cumulative incidence of breast cancer in their study groups and concluded that BSE has a significant effect on early (<Stage 3) detection of breast cancer and therefore is an effective practice that must be propagated at the community level [9]. A recent study among Japanese youth found that a scare of cancer was leading to young adults getting unnecessary frequent mammograms and concluded that there is a need for more BC awareness, even among medical staff, and promotion of BSE as a better practice for young adults [10].

Sources of information regarding BC, including BSE

For the majority of our study participants, their source of introduction to BSE was healthcare staff, with some of the participants reporting social media, books, and family members as their sources of information. Similarly, most female medical students at an African university cited lectures by medical faculty, followed by books and social media [11,12]. In India, the media and the internet were the major sources of information in studies done on health sciences students in Bangalore and Kashmir [13,14]. Among female non-medical university students in Ethiopia, Cameroon, Malaysia, and China, the majority reported mass media, television, newspapers and magazines, medical health personnel, and television as their sources of information [15-18].

Knowledge of BC risk factors and screening recommendations

We found that 67.6% had good knowledge of risk factors for BC. However, very few participants knew the right age at which it was recommended to start BSE, and mammography, and the right frequency of doing them. Similar findings were reported among medical students in Ethiopia [11]. While healthcare professionals have better knowledge, there is still a lot of scope for improvement in their BC knowledge [19,20]. Non-medical students in universities in Ethiopia, Bangladesh, Cameroon, and Pakistan showed poor knowledge and a stark need for more awareness about BC and its risk factors and screening modalities [16,17,21].

The practice of BSE at least once vs. every month regularly

In our study, 81.3% had performed BSE at least once, but only 27.4% of participants reported regularly practicing BSE. Similar results were seen in a study among nursing students in Turkey and medical students in Ethiopia and Ghana [11,12,22]. Contrarily, studies among medical students from Iran and Kashmir and non-medical students from Turkey, Malaysia, and Bangladesh reported lesser percentages of BSE practice [14,15,20,21,23,24].

Factors significantly associated with BSE practice

In this study, type of family, good knowledge of BC risk factors, and being taught the technique of BSE by healthcare staff, family, or other teaching faculty were significantly associated with regular BSE. In a similar vein, studies done in health sciences colleges in Ethiopia, Kashmir, and Turkey found years of university study, family history of BC, and knowledge of BC to be significantly associated with the practice of BSE to significantly improve the chances of performing BSE regularly [11,14,22]. On the contrary, among non-medical university students across the world, apart from good knowledge, age, marital status, having a good attitude towards BSE, being urban residents, and social status were found to be significantly associated with the practice of BSE [15,17,18,21]. Almost all the studies that evaluated the determinants of BSE practice concluded that closing the knowledge gap about BC would facilitate following preventive and screening recommendations.

Barriers and facilitators of BSE

In our study, fear of diagnosis, discomfort with the practice, and a lack of confidence in performing BSE correctly were common barriers among participants who did not practice BSE regularly. While we did not find any previous studies in which IDIs were conducted to explore the barriers and facilitating factors of BSE practice, there were studies in which participants picked a barrier from a multiple-choice question. Barriers put forward by these studies across the world included "lack of privacy," "lack of time," "don't know how to do it," "worry about detecting breast cancer," "shyness," and "not knowing the technique of BSE" [11,15,21,25]. Women who had more knowledge about BC early diagnosis scored significantly higher in health motivation, BSE self-efficacy, and perceived benefits subscales. Notably, the perceived benefits of BSE and the severity of BC were positively associated with the behavior of practicing BSE [12,26-29]. We found peer support to be a facilitator for regular BSE. This was also reported among Iranian women in the medical field [24].

Limitations

Due to the quantitative part of the study being cross-sectional, we could not evaluate the effect of the study on awareness, knowledge, and practice of BSE. Our study participants were all from a single university, so the generalizability of the findings may be limited.

Implications

Medical students have the opportunity to influence and teach not only the general population but also healthcare workers and allied health professionals. Therefore, bridging the knowledge and skill/technique gap among medical students can go a long way toward improving the knowledge, attitude, and practices of females in our nation. Our IDIs successfully started a conversation about BC awareness and the need for young adults to be proactive with preventive practices, which sparked an intention in medical students to become efficient in BSE, regularly practice it, and counsel others for the same.

## Conclusions

This study reveals a gap between knowledge of risk and its translation into the practice of preventive options. While a majority of respondents were aware of BC risk factors and BSE as a screening method, their practice of preventive methods is low, and they lack sound knowledge of recommendations for following these methods. The insights gained from our interviews can be used to design an effective training program for promoting breast cancer preventive health behaviors. Breast cancer awareness sessions and BSE interventions must be designed keeping in mind the inhibitions and motivations of this target population with the objective of filling the knowledge lacunae and bridging the knowledge-to-practice gap of our future healthcare providers.
